# Isolated laryngeal tuberculosis complex infection: expect the unexpected

**DOI:** 10.1093/omcr/omae007

**Published:** 2024-03-25

**Authors:** Giulia C D’Aleo Canova, Chiara Zeroli, Federico Barberi, Armela Gorica, Maurizio Bignami, Augusto Cattaneo

**Affiliations:** Department of Otorhinolaryngology, Hospital of Circolo and Macchi Foundation, Varese, Italy; Department of Otorhinolaryngology, Sant'Anna Hospital, San Fermo della Battaglia, Como, Italy; Department of Otorhinolaryngology, Hospital of Circolo and Macchi Foundation, Varese, Italy; Department of Otorhinolaryngology, Sant'Anna Hospital, San Fermo della Battaglia, Como, Italy; Department of Otorhinolaryngology, Hospital of Circolo and Macchi Foundation, Varese, Italy; Department of Otorhinolaryngology, Sant'Anna Hospital, San Fermo della Battaglia, Como, Italy; Department of Otorhinolaryngology, Hospital of Circolo and Macchi Foundation, Varese, Italy; Department of Otorhinolaryngology, Sant'Anna Hospital, San Fermo della Battaglia, Como, Italy; Department of Otorhinolaryngology, Hospital of Circolo and Macchi Foundation, Varese, Italy; Department of Otorhinolaryngology, Sant'Anna Hospital, San Fermo della Battaglia, Como, Italy; Department of Otorhinolaryngology, Sant'Anna Hospital, San Fermo della Battaglia, Como, Italy

**Keywords:** respiratory disorders, rheumatology, infectious diseases and tropical medicine

## Abstract

Laryngeal tuberculosis (LT), a rare but possible manifestation of extrapulmonary tuberculosis (TBC) and the most frequent granulomatous disease of the larynx, is slowly resurfacing due to the worldwide recrudescence of TBC. We present the case of a 59 y-o Caucasian woman, non-smoker, with no history of recent travels in endemic areas, affected by pulmonary sarcoidosis, that presented with a symptomatic vegetating lesion involving the left free margin of the epiglottic and a small, ulcerated lesion over the right arytenoid mucosa. While the patient’s profile would not lead to a primary suspect of laryngeal TBC, the diagnostic workup and histological examination confirmed the unusual finding, and the patient was started on standard antitubercular therapy, with a complete laryngeal response. Although isolated laryngeal tuberculosis is still a rare finding, it should be kept into consideration also in non-endemic areas, especially in patients with chronic disease requiring immunosuppressive drugs.

## INTRODUCTION

Tuberculosis (TBC), a chronic infectious disease sustained by *Mycobacterium tuberculosis* complex, typically affects the lungs but occasionally occurs in other sites. Its incidence is steadily increasing due to a rise in immunosuppressive diseases, HIV infection, migration flows and the emergence of antituberculous-resistant organisms [1]. Laryngeal tuberculosis complex infection is an infrequent extrapulmonary manifestation, accounting only for 1% of total TBC incidence [2]. A solitary laryngeal involvement is rare but possible: an exophytic or ulcerous lesion may mimic a carcinoma and should also be differentiated from other granulomatous disease of the upper airways. The aim of this report is to present a case of laryngeal tuberculosis in a non-endemic area, in a patient under immune-suppressive treatment for pulmonary sarcoidosis, as reminder to not underestimate the atypical laryngeal TBC manifestations.

## CASE REPORT

A 59 y-o woman with a 2-month sore throat, cough, hoarseness, and weight loss of 10 kg in the last few months was evaluated at the ENT Department of the Sant’Anna Hospital (Como, Italy). She had no history of smoking or drinking but previously underwent a left quadrantectomy for ductal carcinoma and was under treatment with hydroxychloroquine 400 mg/day and oral cortisone 15 mg/day since 5 years for stage II sarcoidosis. The physical examination was unremarkable, and a blood workup revealed both a normal complete blood count and metabolic panel.

**Figure 1:**
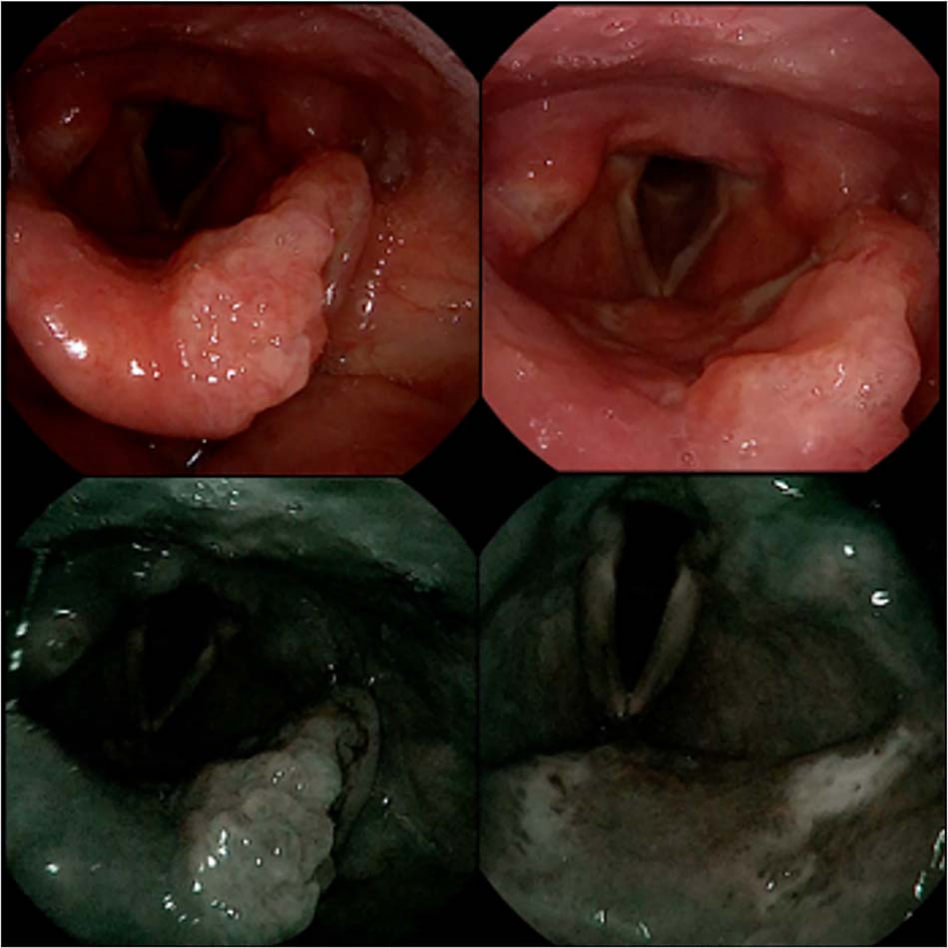
Flexible laryngoscopy with natural and NBI lighting showing a polylobate lesion involving the left free border of the epiglottis (left image) plus other spot lesions on the laryngeal surface of the epiglottis and a millimetric ulcerated area on the right arytenoid mucosa (right image).

A flexible laryngoscopy using white and enhanced lights revealed a centimetric polylobulated lesion involving the free border of the epiglottis with other inframillimetric lesions on its laryngeal surface and a further millimetric ulcerated area on the right arytenoid mucosa (Fig. 1). Vocal folds and arytenoids motility were preserved, and neck examination was unremarkable.

Due to the simultaneous involvement of different laryngeal subsites, in the suspicion of a malignancy, a contrast-enhanced MRI of the neck and a total body PET-CT scan were performed, showing a small, low-enhanced lesion of the epiglottic border, without any other laryngeal enhancements. Multiple ground glass lung masses and bilateral nodules were also reported, increased over previous controls of the known pulmonary sarcoidosis.

**Figure 2:**
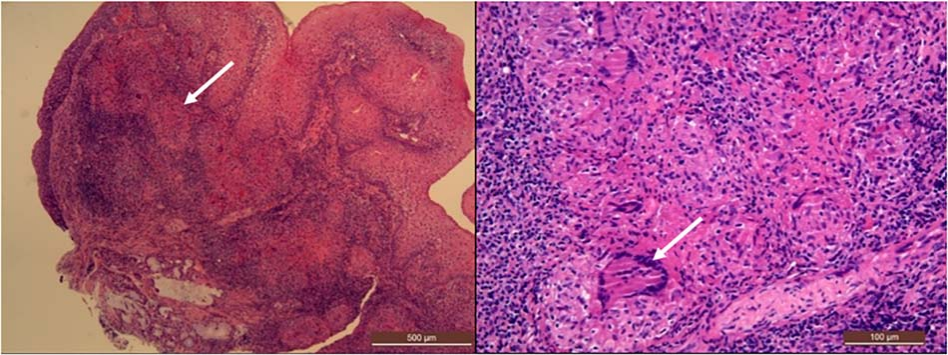
Histologic examination of the laryngeal biopsy shows a pattern of granulomatous inflammation with areas of necrosis diffusely involving the subepithelial stroma, indicated by the white arrows (low and high magnification, respectively 50× and 200×, hematoxylin and eosin stain).

Laryngeal biopsies were performed via direct microlaryngoscopy and the histo-pathological examination described a chronic granulomatous disease with giant cell-associated necrosis of the subepithelial chorion (Fig. 2) and a focal structure morphologically referable to mycobacterium highlighted by histochemical Ziehl Neelsen staining. Due to the histological diagnosis of TBC, the patient also underwent a bronchoalveolar lavage that resulted in a PCR positivity for *Mycobacterium Tuberculosis* complex (MTC), sensitive to rifampicin. She was referred to the infectious disease department and started on antitubercular treatment with rifampicin 600 mg and isoniazid 300 mg once a day (to be taken on an empty stomach), pyrazinamide 500 mg and ethambutol 400 mg 3 times a day plus vitamin B complex. With no history of contact with patients who had cough nor family history of TBC, she was discharged 7 days after admission with the indication to home isolation and 2 months check-up.

**Figure 3:**
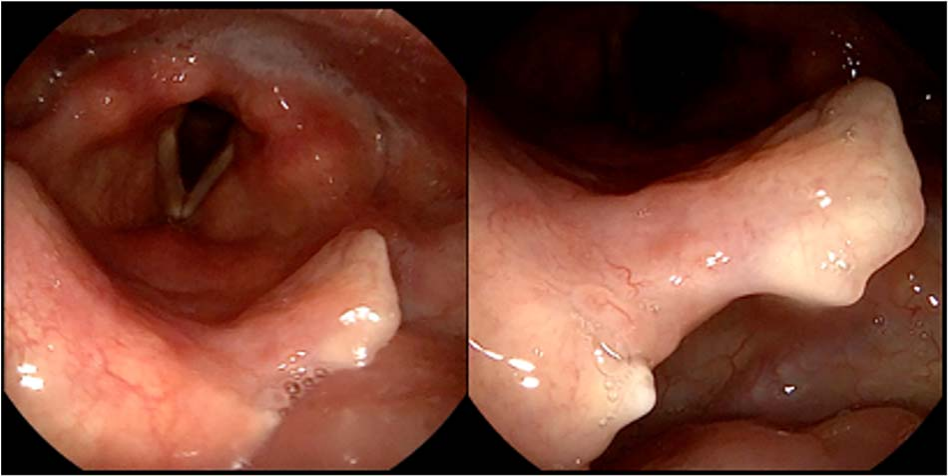
2-months follow-up flexible laryngoscopy, displaying surgical outcomes with a notch on the free margin of the epiglottis and a complete resolution of the mucosal lesions.

As soon as 2 weeks after the start of the treatment, she already reported resolution of previous laryngeal symptoms and cough. A 2 months ENT evaluation highlighted complete disappearance of the laryngeal lesions (Fig. 3.), and a sputum examination resulted negative for MTC. She therefore continued the antitubercular treatment only with rifampicin and isoniazid for another 7 months. A thorax CT scan showed no further growth or variations of the pulmonary findings, therefore indicative of sarcoidosis. The patient then continued the medical treatment with rifampicin and isoniazid and was referred to her community hospital for follow up.

## DISCUSSION

Laryngeal tuberculosis complex infection is a rare condition whose diagnosis requires a high degree of clinical suspicion. Usually developing from advanced pulmonary TBC through bronchogenic, hematogenous, or lymphatic spread [2], in cases of unknown active pulmonary disease, it may simulate, both endoscopically and radiologically, a laryngeal malignancy. Although laryngeal TBC classically presents as diffuse whitish edema, ulcerated lesions or chondritis, recent literature shows an evolution in its clinical characteristics, with hypertrophic or exophytic lesions, therefore increasing the risk of misinterpretation and broadening the spectrum of potential differential diagnosis [3]. Especially in non-endemic areas and in patients who do not fit the typical high-risk profile for laryngeal cancer, there are various granulomatous chronic diseases affecting the larynx to be considered, such as syphilis, sarcoidosis, Wegener granulomatosis and actinomicosis [4, 5].

In our case the patient was affected by pulmonary sarcoidosis, which is diagnosed by associating clinical and radiological findings (thorax CT scan), excluding other disorders and histologically confirming typical granulomas. The severity and extent of the condition determines the type of treatment, ranging from corticosteroids to immunosuppressants such as methotrexate, azathioprine or hydroxychloroquine. Sarcoidosis has a very low incidence of laryngeal involvement, around 0.6%, and usually affects the supraglottic region [6], presenting as diffuse thickening also called “turban-like” epiglottis. It can also manifest as small nodular lesions or localized infiltrative lesions [7]. While laryngeal TBC can either present as dysphonia, odynophagia or dyspnea, sarcoidosis produces hoarseness of voice with a progression to airway obstruction and stridor [8]. Radiological differences between the two are of little to no interest, both presenting as nodular lesions with contrast enhancement and an increased uptake on the PET/CT scan [9, 10]. Due to their inconstant laryngoscopic presentation and clinical-radiological similarities, histological examination is mandatory. In case of laryngeal sarcoidosis, biopsy typically shows multiple non-caseating epithelioid cell granulomas, made of mononuclear cells with a variable degree of necrosis, leucocyte infiltration and fibrosis. Instead, laryngeal TBC will display tuberculous follicle characterized by a peripheral zone of Langhans cells, epithelioid cells and lymphocytes with a central caseous necrosis, and acid-fast bacilli when exposed to Ziehl-Neelsen staining. Histological diagnosis is not always decisive and in cases suggestive for chronic inflammatory disease with a negative acid-fast bacilli, further investigations should be performed, such as a sputum test. Beyond the urge to rule out any malignancy, the effort to distinguish laryngeal TBC from sarcoidosis is mandatory, considering the significantly different therapeutical approaches, with high dose cortisone opposed to long-term antitubercular therapy.

With the global recrudescence of TBC, clinicians should be aware of atypical manifestations even in non-endemic areas, especially in fragile patients such as those undergoing immunosuppressive treatments.
